# Vascular Calcification Patterns in the Elderly: Correlation Between Aortic and Iliac Calcification Burden

**DOI:** 10.3390/diagnostics15243151

**Published:** 2025-12-11

**Authors:** Maximilian Lutz, Malik Galijasevic, Richard A. Lindtner, Andreas E. Ellmerer, David Wippel, Elke R. Gizewski, Stephanie Mangesius, Dietmar Krappinger, Alexander Loizides

**Affiliations:** 1 Department of Radiology, Medical University of Innsbruck, 6020 Innsbruck, Austria; 2 Department of Orthopaedics and Traumatology, Medical University of Innsbruck, 6020 Innsbruck, Austria; 3 Department of Vascular Surgery, Medical University of Innsbruck, 6020 Innsbruck, Austria

**Keywords:** vascular calcification, aging, calcification score, computed tomography

## Abstract

**Background**: Vascular calcification is a frequent consequence of ageing and is associated with an increased risk of cardiovascular disease. This study aimed to compare two rapid scoring systems for quantifying calcification of the distal abdominal aorta and iliac arteries and to investigate correlations with increasing age. **Methods**: Patients aged ≥65 years who sustained pelvic trauma between 2003 and 2023 and underwent computed tomography (CT) were included in this retrospective study. Patients were categorised into three age groups (65–74, 75–84, ≥85). The abdominal aorta calcification score (AACS) and the common, external, and total iliac artery calcification scores (CIACS, EIACS, TIACS) were assessed on cross-sectional images and classified into three severity grades (mild, moderate, severe). **Results**: A total of 224 patients (mean age 78.8 ± 8.5 years; 62% female) were included. Significant differences between age groups were identified for hypertension (*p* < 0.001), osteoporosis (*p* < 0.001), atrial fibrillation (*p* = 0.015), chronic heart failure (*p* = 0.004), chronic kidney disease (*p* < 0.001), neurocognitive disorders (*p* < 0.001), and anticoagulant therapy (*p* = 0.002). Calcification severity increased with age across all vascular territories (EIACS *p* = 0.006; others *p* < 0.001). In multivariable linear regression, age remained the strongest adjusted predictor of calcification across all vascular regions (β = 0.323–0.376, all *p* < 0.001). Significant positive correlations were found between aortic and iliac calcifications (all *p* < 0.001), strongest between AACS and CIACS (ρ = 0.78, CI 0.719–0.835) and TIACS (ρ = 0.745, CI 0.676–0.807). Corresponding categorical associations were most pronounced between AACS and CIACS. **Conclusions**: The evaluated calcification scores were strongly correlated and demonstrated clear age-dependent trends. Given their simplicity and applicability to routine CT imaging, these methods may provide practical tools for assessing vascular ageing.

## 1. Introduction

Vascular calcification is a common age-related phenomenon that contributes substantially to cardiovascular morbidity and mortality in older adults. Its pathogenesis is multifactorial, involving oxidative stress, inflammation, mitochondrial dysfunction, cellular senescence, and diverse biochemical signalling pathways [1,2,3,4,5,6]. Several factors are associated with the development of vascular calcification, including hypertension, diabetes mellitus, chronic kidney disease, increased body mass index, male sex and smoking [5,7,8,9,10,11,12,13,14,15,16,17]. Although vascular calcifications can already be detected in young adults, its prevalence and severity increase markedly with age, which remains the most consistent determinant [8,9,10,11,12].

The clinical consequences of arterial calcification include increased arterial stiffness and pulse wave velocity, as well as impaired Windkessel function of the aorta, ultimately resulting in reduced tissue perfusion and contributing to end-organ damage [4,6,18,19,20]. These pathophysiological changes translate into a higher risk of cardiovascular events and mortality [6,18,21,22,23,24].

Calcifications can affect various vascular territories, with early changes often observed in the coronary arteries, distal abdominal aorta, and iliac arteries [9,11,15]. Although vascular calcification progresses systemically, its distribution and severity vary between territories [9,11,15]. Notably, an electron-beam computed tomography (CT) study by Allison et al. [9] demonstrated a moderate correlation between calcification in the distal aorta and iliac arteries.

Various methods have been proposed for quantifying vascular calcification in CT, ranging from automated algorithms to simpler, semi-quantitative approaches that do not require specialised tools. The objective of this study was to assess the correlation between two rapid and accessible methods for quantifying vascular calcification in the distal abdominal aorta and iliac arteries in elderly individuals. Additionally, we aimed to evaluate the relationship between vascular calcification severity and patient age.

## 2. Materials and Methods

### 2.1. Study Design

This retrospective cohort study included patients aged ≥65 years who were treated for pelvic trauma at a tertiary care centre in Europe between July 2003 and June 2023. The inclusion criterion was the availability of CT imaging that fully depicted the abdominal aorta from the origin of the inferior mesenteric artery to the level of the common femoral arteries. Patient-specific data were reviewed using the local electronic patient record system, while study data were managed using Microsoft Excel (version 2309, Microsoft Corporation, Redmond, WA, USA). The study was approved by the local ethics committee (ethics approval number: EK Nr. 1041/2025).

### 2.2. Assessment of Vascular Calcification

CT datasets were evaluated using multiplanar reconstructions within the DeepUnity Diagnost Picture Archiving and Communication System (PACS) (version 2.0.2.2, Dedalus Healthcare Group, Milan, Italy). Imaging protocols varied among patients, as some scans originated from referring hospitals, and both non-contrast and contrast-enhanced scans were included. Slice thickness differed across examinations, with a maximum of 5 mm. The thinnest available slice thickness was used for analysis.

#### 2.2.1. Abdominal Aortic Calcification

**Figure 1:**
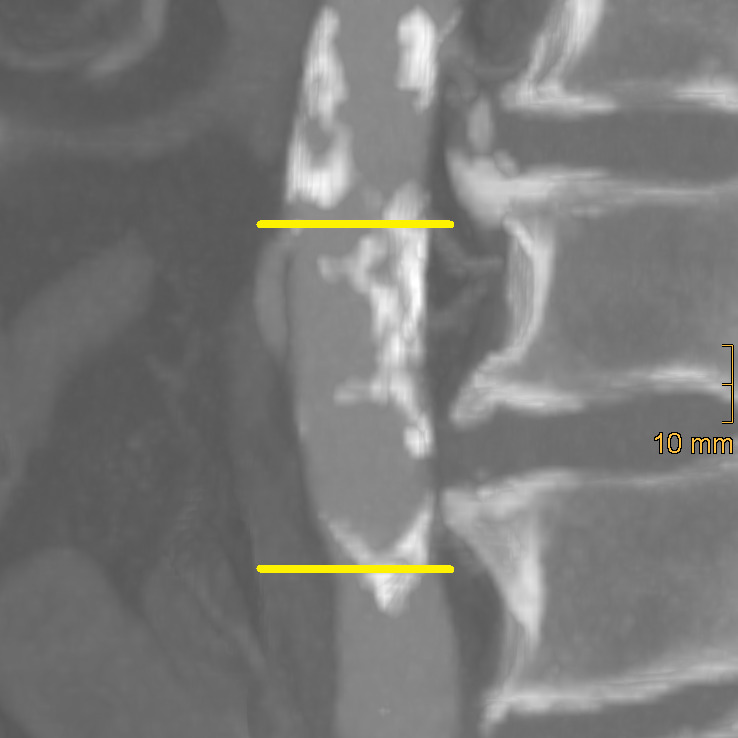
Axis-corrected sagittal reformation of the distal abdominal aorta. The yellow lines mark the origin of the inferior mesenteric artery and the aortic bifurcation. There is only punctual calcification at the anterior wall (=1 point), whereas more than two-thirds of the posterior wall show calcifications (=3 points). Accordingly, the AACS equals 4.

Aortic calcification was assessed using the method described by Reddy et al. [25], evaluating the segment between the origin of the inferior mesenteric artery and the aortic bifurcation. First, multiplanar reformations were generated to correct for aortic elongation. These were subsequently reviewed in sagittal orientation. To further enhance the visualisation of calcifications, 20 mm maximum intensity projections were created. Final image assessment was performed in a standard bone window (window width = 2500 HU; window level = 300 HU). Both the anterior and posterior walls were scored individually from 0 to 3 based on the longitudinal extent of calcification (0 = none, 1 = <33%, 2 = 33–66%, 3 = >66%). The total abdominal aortic calcification score (AACS) ranged from 0 to 6 and was classified as 0–2 = none to mild, 3–4 = moderate, 5–6 = severe. An example of AACS classification is provided in Figure 1.

#### 2.2.2. Iliac Artery Calcification

**Figure 2:**
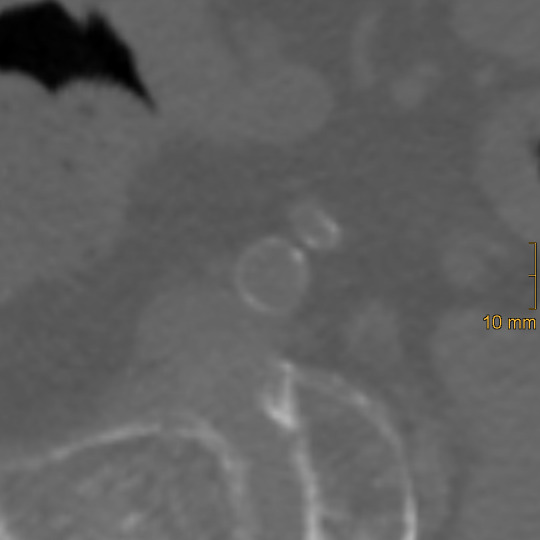
Example of thin linear calcification in the left common iliac artery, corresponding to a morphology score of 1.

**Figure 3:**
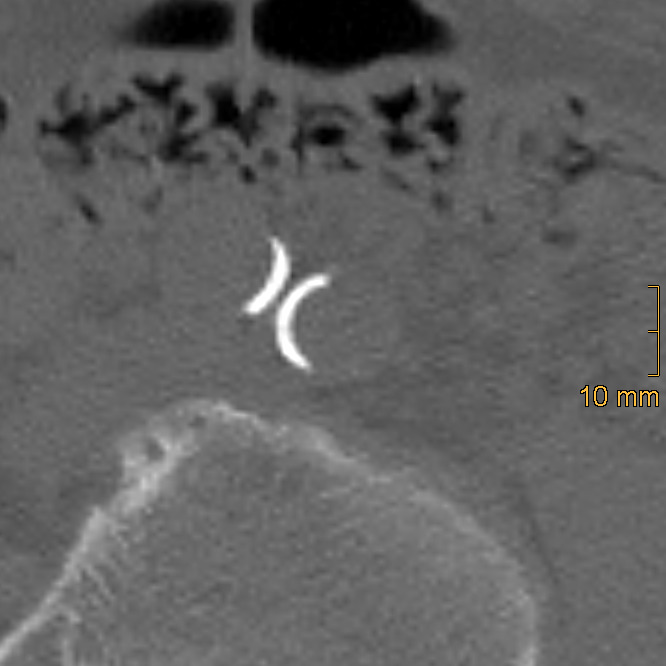
Example of thick linear calcification in the left common iliac artery, corresponding to a morphology score of 2. An additional thick linear calcification is also visible in the right common iliac artery.

**Figure 4:**
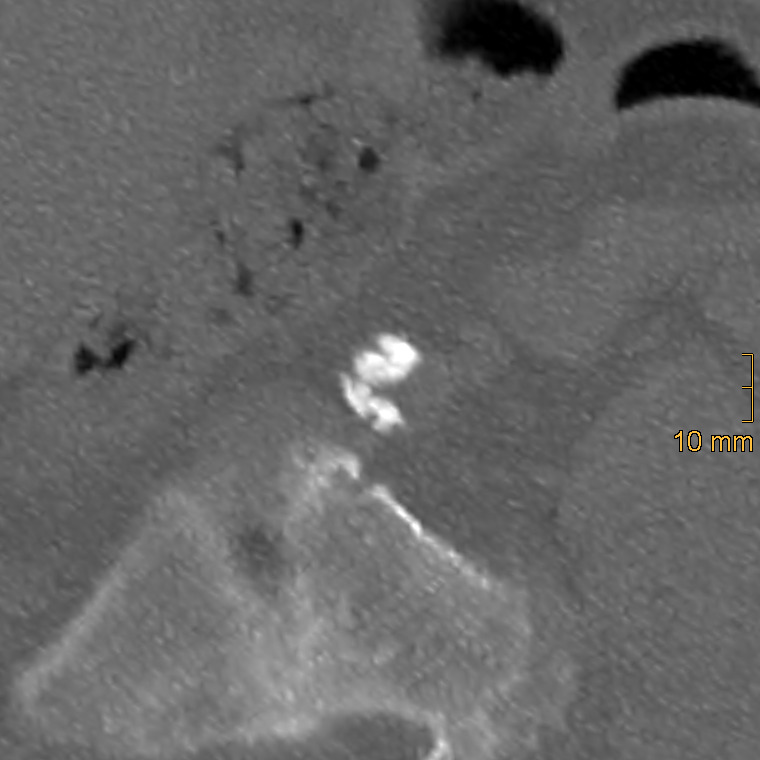
Example of a thick, bulky calcification with convex luminar margins in the left common iliac artery, corresponding to a morphology score of 3.

Iliac calcification was assessed using a semi-quantitative method described by Davis et al. [26]. Both common iliac arteries (CIA) and external iliac arteries (EIA) were evaluated bilaterally using standard bone window settings (window width = 2500 HU; window level = 300 HU). The CIA was defined from the aortic bifurcation to the iliac bifurcation, and the EIA from the iliac bifurcation to the level of the superior aspect of the femoral head. Each vessel segment was scored in three domains: morphology (0–3), circumference (0–4), and length of involvement (0–4). To ensure accurate assessment, multiplanar reformations were generated to correct for vessel tortuosity and to obtain orthogonal cross-sectional views, which is essential for the evaluation of calcification morphology and circumferential extent, whereas the longitudinal extent of calcification was assessed on standard axial images. Morphology was graded as follows: 0 = no calcifications, 1 = thin linear (≤1 mm), 2 = thick linear (>1 mm), 3 = bulky (>2 mm, convex luminal margins). Representative examples of morphologic grading are shown in Figure 2, Figure 3 and Figure 4. Circumference and length of involvement were scored as 0 = no calcifications, 1 = 1–25%, 2 = 26–50%, 3 = 51–75%, 4 = 76–100%. The highest score per segment was used in cases of multiple calcified regions. For each patient, bilateral scores were summed and averaged, resulting in the common iliac artery calcification score (CIACS) and the external iliac artery calcification score (EIACS). Both scores were subsequently combined to generate the total iliac artery calcification score (TIACS). Severity was classified as follows: 0–3.75 = none to mild, 4–7.75 = moderate, 8–11 = severe.

All measurements were performed by a radiology resident with three years of experience.

### 2.3. Statistical Analysis

Statistical analyses were performed using IBM SPSS Statistics (Version 30.0, IBM Corp., Armonk, NY, USA). Raw scores were obtained for the abdominal aorta, CIA, EIA, and the combined iliac territory. Age was categorised into three groups: 65–74, 75–84, and ≥85 years.

The Shapiro–Wilk test was used to assess the normality of continuous variables. Descriptive statistics are presented as means ± standard deviation for normally distributed continuous variables or medians with interquartile ranges (IQR) for non-normally distributed data, and as frequencies with percentages for categorical variables. Associations between categorical variables were analysed using Pearson’s Chi-squared test, and effect sizes were reported using Cramer’s-V. Post hoc pairwise comparisons between age groups were adjusted using the Bonferroni correction. Spearman’s rank correlation coefficients (ρ) were calculated to evaluate associations between continuous calcification scores. To assess the precision of correlation estimates, 95% confidence intervals (CI) were obtained using non-parametric bootstrapping with 1000 samples (bias-corrected and accelerated method). Multivariable linear regression analyses were performed to assess the independent associations between calcification scores, age, and comorbidities.

Statistical significance was defined as *p* < 0.05 (two-tailed). Results were visualised using proportionally stacked bar plots; non-significant visualisations were omitted for clarity.

## 3. Results

### 3.1. Patient Characteristics

A total of 224 patients were included, of whom 139 (62%) were female. The mean age was 78.8 ± 8.5 years. The most common comorbidities were hypertension (54.0%) and osteoporosis (36.2%). Further demographic details are presented in Table 1.

Post hoc analyses with Bonferroni correction revealed that the prevalence of hypertension and anticoagulant medication was significantly higher in both older groups compared to patients <75 years, while there was no significant difference between the two older groups. Osteoporosis, chronic kidney disease, and neurocognitive disorders were significantly more frequent in patients ≥ 85 compared with both younger groups, whereas the prevalence of heart failure and atrial fibrillation differed only between the youngest and the oldest patients.

### 3.2. Distribution of Vascular Calcification

**Figure 5:**
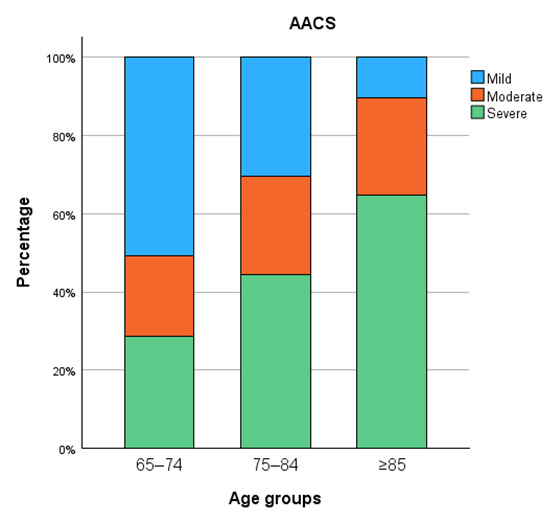
Aortic calcification severity.

**Figure 6:**
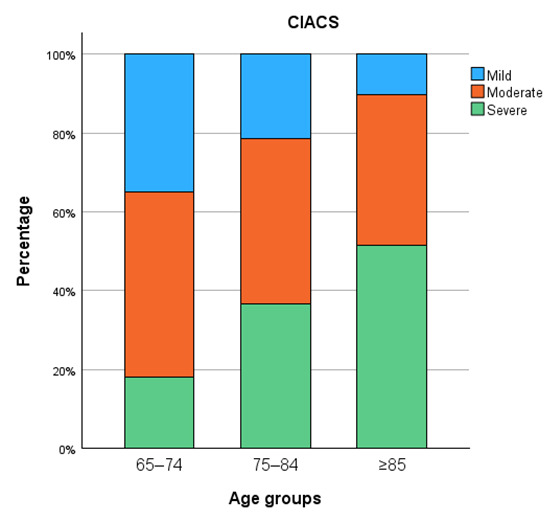
Common iliac artery calcification severity.

**Figure 7:**
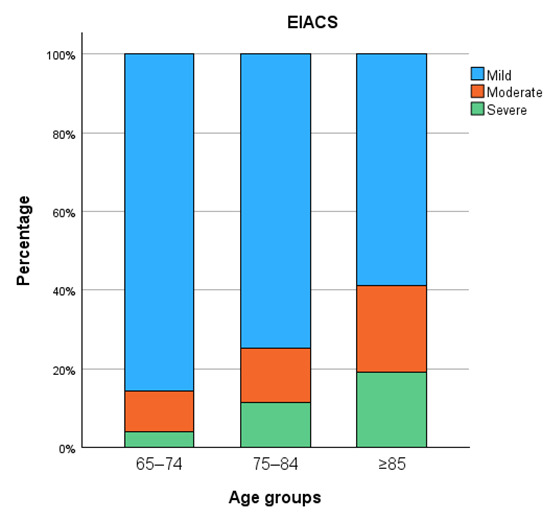
External iliac artery calcification severity.

**Figure 8:**
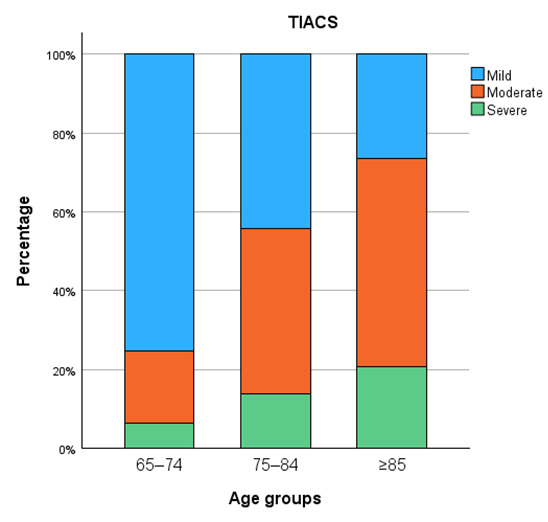
Combined iliac artery calcification severity.

Calcification severity increased progressively with age across all vascular territories (Figure 5, Figure 6, Figure 7 and Figure 8). In the distal abdominal aorta, the median AACS was 4 (IQR = 4), reflecting predominantly moderate calcification with a clear trend towards higher severity among patients aged ≥85 years. In the CIA, the distribution pattern was comparable, with the most pronounced increase observed within the moderate category (median CIACS = 6.5, IQR = 4.5). In contrast, calcifications in the EIA were mostly mild, observed in 79% of patients, with a median score of 1.5 (IQR = 4). The TIACS again demonstrated a shift towards moderate severity with a median of 4 (IQR = 4.25). Detailed score distributions are presented in Table 2.

Pairwise post hoc analyses with Bonferroni correction revealed that aortic calcification severity was significantly higher in patients aged ≥85 years compared with both younger age groups, whereas CIACS and EIACS differed significantly only between the youngest and the oldest groups. TIACS severity was significantly higher in both older age groups compared with patients aged 65–74 years but did not differ significantly between the two older groups.

### 3.3. Correlation and Categorical Associations of Vascular Calcification

Spearman’s rank correlation analysis demonstrated significant positive associations between continuous variables across all calcification sites (all *p* < 0.001). The AACS correlated strongly with CIACS (ρ = 0.78, CI 0.719–0.835) and TIACS (ρ = 0.745, CI 0.676–0.807), while the correlation between AACS and EIACS was moderate (ρ = 0.573, CI 0.469–0.668). The strongest correlations were observed between TIACS and CIACS (ρ = 0.945, CI 0.925–0.960) and TIACS and EIACS (ρ = 0.89, CI 0.851–0.920), with a strong correlation also noted between CIACS and EIACS (ρ = 0.724, CI 0.646–0.787).

There were significant associations between calcification categories across all vascular territories (all *p* < 0.001). The strongest associations were found between TIACS and EIACS (χ^2^ = 216.7, df = 4, Cramer’s V = 0.695), TIACS and CIACS (χ^2^ = 151.9, df = 4, Cramer’s V = 0.582) and AACS and CIACS (χ^2^ = 128.3, df = 4, Cramer’s V = 0.535). Additional information regarding categorical associations is presented in Table 3.

### 3.4. Multivariable Linear Regression Analysis

Multivariable linear regression analyses were performed with continuous calcification scores as dependent variables and included age, sex, hypertension, osteoporosis, chronic heart failure, chronic kidney disease, neurocognitive disorders, and antiplatelet therapy as independent variables. In these models, age remained the strongest predictor of all four calcification scores (AACS, CIACS, EIACS, TIACS) after adjustment for all covariates (all *p* < 0.001). Chronic kidney disease showed an association with higher AACS values after adjustment (*p* = 0.010), whereas heart failure was associated with higher EIACS values (*p* = 0.003). Antiplatelet therapy was independently associated with higher calcification scores across all vascular territories (*p* = 0.005–0.022). A detailed overview of the analyses is presented in Table 4, Table 5, Table 6 and Table 7.

## 4. Discussion

In this retrospective study of elderly patients with pelvic trauma, we evaluated two rapid and pragmatic scoring methods for quantifying vascular calcification in the distal abdominal aorta and iliac arteries. We found strong associations between the different calcification scores, particularly between AACS and CIACS. Furthermore, the severity of vascular calcification increased significantly with age across all vascular territories. These results suggest that both scoring methods reliably reflect the burden of vascular calcification and may serve as efficient tools for risk stratification in older populations.

Several methods have been described for quantifying aorto-iliac vascular calcifications [27]. For the assessment of the distal abdominal aorta, we applied the AACS as described by Reddy et al. [25]. This scoring system provides a fast and reproducible method for quantifying distal aortic calcifications, demonstrating excellent interobserver agreement and strong correlation with calculated calcium scores [25]. In the present study, we divided the total score into three severity levels to classify patients based on their calcification burden. Thresholds were defined at nearly equal intervals to ensure a consistent grading scale across all categories.

For iliac artery calcification, we adopted the method initially introduced by Davis et al. [26], which was further adapted by Huynh et al. [24]. While maintaining the core structure of this method, our approach incorporated several modifications. Calcification scores were averaged across bilateral vessels to reduce variability, to avoid excessive score inflation that would result from pure summation, and to allow side-independent comparisons between patients. A potential downside of this approach is that it may obscure asymmetric calcification patterns or unilateral high-grade stenosis. Based on the CIACS and EIACS, we constructed the TIACS to reflect the calcification burden of the iliac axis. Calcification of the internal iliac artery was not assessed due to limited scoring reliability and therefore did not contribute to the TIACS. It must be acknowledged that the TIACS has not been validated against clinical outcome parameters and should therefore be regarded as an exploratory score. Unlike Huynh et al. [24], who applied narrower and uneven threshold ranges for iliac artery calcification scoring, we defined almost evenly spaced cut-off values. This decision was made to ensure consistent discrimination across severity categories and to maintain comparability with the classification scheme used for the AACS.

The main advantage of these pragmatic scoring systems lies in their straightforward application to routine CT datasets. They were intentionally selected for this study to enable rapid and reproducible estimation of vascular calcification burden directly within a standard PACS environment, with the assessment of the respective scores requiring only a few minutes. In contrast to automated or algorithm-based approaches, these systems do not require specialised software or additional computational steps. Furthermore, there are no additional costs associated with their use. Despite these advantages, it has to be acknowledged that automated algorithms are currently the subject of intensive research and hold immense potential for providing both qualitative and quantitative information on vascular calcification. Future studies should therefore compare these simple, semi-quantitative methods with automated, algorithm-based approaches to further assess their relative accuracy and robustness. In addition to CT-based approaches, X-ray-based quantification systems like the 24-point score initially described by Kauppila et al. [28] or the simplified 8-point score described by Schousboe et al. [29] are widely used for the assessment of abdominal aortic calcification on lateral lumbar spine radiographs. These systems share important advantages with the approaches used in our study, as lumbar spine X-rays are widely available, particularly in older adults, and the respective scores can be obtained rapidly. When comparing the effectiveness of vascular calcification detection, NasrAllah et al. [30] demonstrated that CT-based techniques are more sensitive than plain radiographs for detecting aortic and peripheral vascular calcification. These differences are likely related to the absence of superimposition effects in CT imaging, such as from osteophytes or bowel gas, which may limit radiographic assessment. Furthermore, multiplanar reformations in CT allow correction for vessel elongation and offer greater potential for plaque characterisation. As CT imaging is commonly performed in older adults for numerous clinical indications, CT-based scoring systems should therefore be preferred over radiograph-based approaches whenever appropriate CT imaging data are available.

The cohort of this study consisted of elderly patients who suffered pelvic trauma. Our analysis demonstrated an age-related increase in calcification severity across all vascular territories, consistent with previous research identifying age as a dominant determinant of vascular calcification [8,9,10,11,12]. While this relationship is well established, our data confirm that it is also robustly reflected by the rapid scoring systems evaluated in this study. In addition, we identified statistically significant differences in several pre-existing medical conditions between age groups, including hypertension, osteoporosis, atrial fibrillation, chronic heart failure, chronic kidney disease, and neurocognitive disorders, with mild cognitive impairment and various forms of dementia being the main contributors to this entity. All of these conditions are well known to increase with advancing age [31,32,33,34,35,36,37]. The associations with osteoporosis and chronic kidney disease are of particular interest, as both conditions have been linked to vascular calcification through complex mechanisms involving disturbances in calcium-phosphate homeostasis, inflammation, oxidative stress, and osteogenic signalling within the vessel wall [3,5,38,39,40,41,42]. Multivariable linear regression analyses further confirmed that age was the strongest predictor of vascular calcification across all territories after adjustment for clinical covariates. Chronic kidney disease was additionally associated with higher AACS values, whereas chronic heart failure was associated with higher EIACS values. In contrast, several other comorbidities that differed between age groups did not show associations after adjustment. The use of antiplatelet therapy was also associated with calcification burden across all vascular territories. However, this finding most likely reflects the fact that antiplatelet agents are regarded as standard treatment in patients with advanced vascular disease. Beyond the correlations between vascular calcification, age, and comorbidities, the relationship between calcification burden and cardiovascular outcomes remains of clinical relevance. Several studies, especially in patients with vascular disease, have demonstrated that a higher degree of vascular calcification is associated with increased long-term mortality and a greater risk of adverse cardiovascular events [21,22,23,24,43,44,45]. However, in our cohort, data on long-term cardiovascular outcomes were insufficient, precluding a reliable analysis of these associations.

Our findings reinforce the observations made by Allison et al. [9], emphasising the relationship between distal aortic and iliac calcification, and demonstrate that these associations can also be detected using efficient, CT-based scoring approaches. We observed highly significant associations between these territories, indicating that calcification in the distal aorta and iliac arteries progresses in a coordinated pattern across adjacent vascular regions. Patients with severe aortic calcification were markedly more likely to exhibit severe CIACS, whereas mild aortic calcification predominantly aligned with mild or moderate calcification at the CIA. In contrast, the EIA showed overall lower calcification severity, potentially reflecting anatomical or haemodynamic differences between territories. The TIACS, by nature, yielded intermediate values between the CIA and EIA scores and maintained a strong association with aortic calcification, suggesting a continuous distribution of calcification along the aorto-iliac axis.

Although the scoring systems used in this study were primarily designed for research purposes, they may also provide additional information for clinical practice by supplementing descriptive findings with semi-quantitative data. A potential implication of their complementary use could lie in risk stratification of access vessels for endovascular procedures, since these minimally invasive interventions frequently require catheterisation of the iliac axis, and a high calcification burden may complicate this process. However, further studies linking these scores to clinical outcomes and therapeutic decision pathways are required before their role in clinical practice can be clearly defined.

This study has several limitations. First, the study cohort consisted exclusively of trauma patients, who may not accurately represent the general elderly population, thereby potentially limiting the generalisability of our findings. Moreover, the relatively small sample size may have affected the statistical power of the analyses and limited the ability to detect subtle differences between subgroups. Another limitation, partly related to the composition of the study population, is that some patients were transferred to our centre solely for trauma management and continued follow-up elsewhere. Consequently, clinical endpoints such as cardiovascular events or long-term mortality were not fully available and were therefore not evaluated. Furthermore, imaging protocols varied, as CT scans were partly obtained from external institutions, potentially affecting image quality. Finally, all measurements were performed by a single radiologist, which did not allow assessment of interobserver variability.

## 5. Conclusions

In summary, the scoring systems applied to the distal abdominal aorta and iliac arteries demonstrated strong associations and clear age-related trends in this elderly cohort. These findings support their value as accessible and efficient tools for the semi-quantitative assessment of vascular calcification, primarily within research settings. Potential clinical implications may exist, but further validation is required before these methods can be recommended for routine clinical use.

## Figures and Tables

**Table 1:** Basic demographics.

	Cohort *n* = 224	65–74 *n* = 77	75–84 *n* = 79	≥85 *n* = 68	*p*-Value
Age—years	78.8 (8.5)	69.2 (2.9)	79.5 (2.9)	89.0 (3.3)	
Sex—Female	139 (62.1)	39 (50.6)	45 (57.0)	55 (80.9)	<0.001
Hypertension	121 (54.0)	27 (35.1)	51 (64.6)	43 (63.2)	<0.001
Osteoporosis	81 (36.2)	15 (19.5)	25 (31.6)	41 (50.6)	<0.001
CAD	52 (23.2)	13 (16.9)	20 (25.3)	19 (27.9)	0.249
AF	47 (21.0)	10 (13.0)	15 (19.0)	22 (32.4)	0.015
CHF	29 (12.9)	4 (5.2)	9 (11.4)	16 (23.5)	0.004
Stroke/TIA	27 (12.1)	5 (6.5)	9 (11.4)	13 (19.1)	0.065
CKD	26 (11.6)	3 (3.9)	6 (7.6)	17 (25.0)	<0.001
NCD	26 (11.6)	2 (2.6)	5 (6.3)	19 (27.9)	<0.001
DM	19 (8.5)	4 (5.2)	9 (11.4)	6 (8.8)	0.378
Antiplatelet therapy	77 (34.4)	21 (27.3)	29 (36.7)	27 (39.7)	0.25
Anticoagulant therapy	54 (24.1)	8 (10.4)	23 (29.1)	23 (33.8)	0.002

Data are presented as mean (standard deviation) or absolute frequencies (percent of each age group). CAD = Coronary artery disease; AF = Atrial fibrillation; CHF = Chronic heart failure; TIA = Transient ischaemic attack; CKD = Chronic kidney disease; NCD = Neurocognitive disorders; DM = Diabetes mellitus.

**Table 2:** Age-dependent vascular calcification.

	Cohort *n* = 224	65–74 *n* = 77	75–84 *n* = 79	≥85 *n* = 68	*p*-Value
AACS					<0.001
- Mild	70 (31.3)	39 (50.6)	24 (30.4)	7 (10.3)	
- Moderate	53 (23.7)	16 (20.8)	20 (25.3)	17 (25.0)	
- Severe	101 (45.1)	22 (28.6)	35 (44.3)	44 (64.7)	
CIACS					<0.001
- Mild	51 (22.8)	27 (35.1)	17 (21.5)	7 (10.3)	
- Moderate	95 (42.4)	36 (46.8)	33 (41.8)	26 (38.2)	
- Severe	78 (34.8)	14 (18.2)	29 (36.7)	35 (51.5)	
EIACS					0.006
- Mild	165 (73.7)	66 (85.7)	59 (74.7)	40 (58.8)	
- Moderate	34 (15.2)	8 (10.4)	11 (13.9)	15 (22.1)	
- Severe	25 (11.2)	3 (3.9)	9 (11.4)	13 (19.1)	
TIACS					<0.001
- Mild	111 (49.6)	58 (75.3)	35 (44.3)	18 (26.5)	
- Moderate	83 (37.1)	14 (18.2)	33 (41.8)	36 (52.9)	
- Severe	30 (13.4)	5 (6.5)	11 (13.9)	14 (20.6)	

Data are presented as absolute frequencies (percent of each age group). AACS = Abdominal aortic calcification score; CIACS = Common iliac artery calcification score; EIACS = External iliac artery calcification score; TIACS = Total iliac artery calcification score.

**Table 3:** Associations between calcification categories.

	χ^2^ (df = 4)	*p*-Value	Cramer’s V
AACS and CIACS	128.3	<0.001	0.535
AACS and EIACS	38.35	<0.001	0.293
AACS and TIACS	101.26	<0.001	0.475
CIACS and EIACS	81.29	<0.001	0.426
CIACS and TIACS	151.89	<0.001	0.582
EIACS and TIACS	216.66	<0.001	0.695

χ^2^ = Chi-squared; df = Degrees of freedom; AACS = Abdominal aortic calcification score; CIACS = Common iliac artery calcification score; EIACS = External iliac artery calcification score; TIACS = Total iliac artery calcification score.

**Table 4:** Multivariable linear regression analysis for AACS.

Variables	B	SE	β	95% CI	*p*-Value
Age	0.074	0.016	0.323	0.042–0.106	<0.001
Sex (male)	0.199	0.266	0.049	−0.325–0.723	0.455
Hypertension	−0.029	0.248	−0.008	−0.518–0.459	0.905
Osteoporosis	0.488	0.278	0.120	−0.059–1.036	0.080
CHF	−0.214	0.362	−0.037	−0.928–0.500	0.556
CKD	1.004	0.387	0.164	0.242–1.766	0.010
NCD	0.294	0.394	0.048	−0.482–1.070	0.456
Antiplatelet therapy	0.587	0.253	0.142	0.089–1.086	0.021

Model fit: R^2^ (Coefficient of determination) = 0.230; Adjusted R^2^ = 0.201; *p* < 0.001. B = Unstandardized regression coefficient; SE = Standard error; β = Standardized regression coefficient; CI = Confidence interval; CHF = Chronic heart failure; CKD = Chronic kidney disease; NCD = Neurocognitive disorders.

**Table 5:** Multivariable linear regression analysis for CIACS.

Variables	B	SE	β	95% CI	*p*-Value
Age	0.126	0.025	0.358	0.077–0.175	<0.001
Sex (male)	0.200	0.410	0.032	−0.607–1.008	0.625
Hypertension	−0.395	0.382	−0.066	−1.149–0.358	0.302
Osteoporosis	0.569	0.428	0.091	−0.275–1.413	0.185
CHF	0.225	0.559	0.025	−0.876–1.326	0.687
CKD	0.930	0.596	0.100	−0.244–2.105	0.120
NCD	0.323	0.607	0.035	−0.874–1.520	0.595
Antiplatelet therapy	0.899	0.390	0.143	0.130–1.668	0.022

Model fit: R^2^ (Coefficient of determination) = 0.217; Adjusted R^2^ = 0.188; *p* < 0.001. B = Unstandardized regression coefficient; SE = Standard error; β = Standardized regression coefficient; CI = Confidence interval; CHF = Chronic heart failure; CKD = Chronic kidney disease; NCD = Neurocognitive disorders.

**Table 6:** Multivariable linear regression analysis for EIACS.

Variables	B	SE	β	95% CI	*p*-Value
Age	0.123	0.026	0.332	0.071–0.174	<0.001
Sex (male)	0.451	0.428	0.069	−0.393–1.294	0.293
Hypertension	−0.400	0.399	−0.063	−1.186–0.386	0.318
Osteoporosis	0.095	0.447	0.015	−0.786–0.976	0.831
CHF	1.764	0.583	0.188	0.615–2.913	0.003
CKD	0.559	0.622	0.057	−0.667–1.785	0.370
NCD	0.648	0.634	0.066	−0.601–1.898	0.307
Antiplatelet therapy	1.128	0.407	0.170	0.326–1.931	0.006

Model fit: R^2^ (Coefficient of determination) = 0.230; Adjusted R^2^ = 0.202; *p* < 0.001. B = Unstandardized regression coefficient; SE = Standard error; β = Standardized regression coefficient; CI = Confidence interval; CHF = Chronic heart failure; CKD = Chronic kidney disease; NCD = Neurocognitive disorders.

**Table 7:** Multivariable linear regression analysis for TIACS.

Variables	B	SE	β	95% CI	*p*-Value
Age	0.124	0.023	0.376	0.079–0.170	<0.001
Sex (male)	0.326	0.376	0.056	−0.415–1.066	0.387
Hypertension	−0.397	0.350	−0.070	−1.088–0.293	0.258
Osteoporosis	0.332	0.392	0.057	−0.441–1.106	0.398
CHF	0.995	0.512	0.119	−0.015–2.004	0.053
CKD	0.745	0.546	0.085	−0.332–1.822	0.174
NCD	0.486	0.557	0.055	−0.611–1.583	0.384
Antiplatelet therapy	1.014	0.358	0.171	0.309–1.719	0.005

Model fit: R^2^ (Coefficient of determination) = 0.256; Adjusted R^2^ = 0.228; *p* < 0.001. B = Unstandardized regression coefficient; SE = Standard error; β = Standardized regression coefficient; CI = Confidence interval; CHF = Chronic heart failure; CKD = Chronic kidney disease; NCD = Neurocognitive disorders.

## Data Availability

The data, analytic methods, and study materials will be readily made available upon reasonable request addressed to the corresponding author. This approach was chosen in consideration of safety and legal requirements.

## Abbreviations

The following abbreviations are used in this manuscript:

AACS	Abdominal aorta calcification score
AF	Atrial fibrillation
CT	Computed tomography
CAD	Coronary artery disease
CHF	Chronic heart failure
CIA	Common iliac artery
CIACS	Common iliac artery calcification score
CKD	Chronic kidney disease
df	Degrees of freedom
DM	Diabetes mellitus
EIA	External iliac artery
EIACS	External iliac artery calcification score
IQR	Interquartile range
NCD	Neurocognitive disorder
PACS	Picture Archiving and Communication System
TIA	Transient ischaemic attack
TIACS	Total iliac artery calcification score
